# Brief report: risk stratification following curative therapy for stage I NSCLC

**DOI:** 10.3389/fonc.2023.1250315

**Published:** 2023-08-14

**Authors:** Emily Butts, Denise Gococo-Benore, Tanmayi Pai, Muhamad Alhaj Moustafa, Fei Heng, Ruqin Chen, Yujie Zhao, Rami Manochakian, Yanyan Lou

**Affiliations:** ^1^ Department of Internal Medicine, Mayo Clinic Jacksonville, Jacksonville, FL, United States; ^2^ Department of Hematology and Oncology, Mayo Clinic Jacksonville, Jacksonville, FL, United States; ^3^ Department of Mathematics and Statistics, University of North Florida, Jacksonville, FL, United States

**Keywords:** resection, surveillance, recurrence, second primary lung cancer, imaging

## Abstract

**Introduction:**

Surveillance with computed tomography (CT) imaging following curative treatment of stage I non-small cell lung cancer (NSCLC) is important to identify recurrence or second primary lung cancers (SPLC). The pattern and risks of recurrence following curative therapy and optimal duration of surveillance scans remain unknown. The objective of our study is to assess the pattern of recurrence and development of SPLC to risk stratify patients with stage I NSCLC following curative therapy.

**Methods:**

We identified 261 patients who received curative therapy for stage I NSCLC at Mayo Clinic Florida. Data was collected on clinical and demographic features including gender, smoking history, stage, treatment, histologic subtype, and tumor grade. Kaplan-Meier method was used to evaluate the disease free survival (DFS). Cox proportional hazard model was used to identify risk factors for recurrence.

**Results:**

Negative tobacco history and stage IA tumors were associated with significantly prolonged DFS after adjusting for co-variates (p=0.001 and p=0.005). Univariate Cox proportional hazards model identified tobacco history and stage 1B as risk factors for recurrence with unadjusted hazard ratio (HR) of 2.8 and 2.0, respectively. After adjusting for covariates, only stage IB was statistically significant predictor of recurrence with a hazard ratio of 2.1 (Confidence Interval (CI) 95% 1.2-3.6; p=0.007).

**Conclusions:**

An individualized approach that considers risk factors of stage and smoking history may be useful in determining whether to continue annual CT surveillance after five years post curative therapy for stage I NSCLC.

## Introduction

Lung cancer is the leading cause of cancer-related deaths globally, with non-small cell lung cancer (NSCLC) accounting for 85% of lung cancer cases. The majority of patients with NSCLC present with locally advanced or metastatic disease at the time of diagnosis. For patients with stage I NSCLC, the prognosis is encouraging, with a 5-year survival of 70-90% (1). Lobectomy remains the standard of care for these patients (2). Stereotactic body radiation therapy (SBRT) or ablation is considered in patients who are medically unfit for surgery (2, 3). SBRT and ablation are additionally known to provide meaningful benefit in short-term outcomes over surgery in healthy patients (4).

Surveillance following curative treatment is important to monitor for recurrence or a secondary primary lung cancer (SPLC). Lou et al. found that in a cohort of 1294 patients with early-stage lung cancer who underwent surgical resection, recurrences developed in 20% of patients, and SPLC developed in 7% of patients within a 5-year surveillance period (5). Current National Comprehensive Cancer Network (NCCN) guidelines recommend computed tomography (CT) of the chest every 6 months for 2-3 years, followed by CT annually (2). There is no clear guidance on how long to continue CT surveillance, with practice varying widely among providers, and there have been few studies evaluating the risk of recurrence (5–7). The objective of this study is to characterize the patterns and risk factors of lung cancer recurrence post-curative therapy to improve the clinical decision-making of surveillance imaging.

## Methods

### Patient and study details

This study was approved by the institutional review board at Mayo Clinic Florida. A total of 493 patients diagnosed with lung cancer at our institution between 1995-2017 were identified. Patients were included if diagnosed with pathologically proven stage IA or IB of NSCLC using the seventh edition of the American Joint Committee on Cancer and had curative-intent therapy with surgery, radiation therapy, or ablation (8). Patients were excluded if any of the following applied: lack of follow up visit, no surveillance imaging available for review, two primary lung lesions at diagnosis, or deceased within 1 month of curative therapy. In total, 261 patients were included in our analysis.

### Data collection

Data was collected on patient clinical and demographic features, surveillance imaging, pathology, time to recurrence or SPLC, location of recurrence, treatment of recurrence or SPLC, and disease free survival (DFS). Three thoracic oncologists independently reviewed the data to confirm the identification of recurrence or SPLC. Recurrences were defined if same histology and not present at the time of diagnosis of primary NSCLC. SPLC was defined if different histology or present at the time of diagnosis of primary NSCLC.

### Statistical analysis

2399697DFS was evaluated using the Kaplan-Meir method. DFS was defined as alive without recurrence or SPLC. Log-rank tests compared DFS curves between groups. The prognostic associations between risk factors and stage I NSCLC were examined using Cox proportional hazard models.

## Results

### Baseline patient characteristics

2399697In this population-based cohort study of 261 patients with stage I NSCLC, the average age was 72 years (**Table 1**). The majority of patients identified as white (94.6%) and smokers (86.2%). stage IA disease was diagnosed in 207 patients, and stage 1B was diagnosed in 54 patients. Most patients were treated with lobectomy (67%). Segmentectomy or wedge resection was done in 21% patients, and pneumonectomy was performed in 1% of patients. Fewer patients were treated with SBRT (9%) or ablation (2%). Most of the patients (92%) were found to have adenocarcinoma or squamous histology.

**Table 1:** Baseline characteristics and treatment modality of 261 patients with stage 1 NSCLC.

Characteristic	No. (%)
Sex	
Male	129 (49.4)
Female	132 (50.6)
**Age at Diagnosis (years, mean ± SD)**	72 ± 8.4
Race	
White	247 (94.6)
Black	7 (2.7)
Hispanic	3 (1.1)
Asian	1 (0.4)
Unknown	3 (1.1)
Tobacco History	
Current or Former Smoker	225 (86.2)
Non-smoker	35 (13.4)
Unknown	1 (0.4)
Stage	
1A	207 (79.3)
1B	54 (20.7)
Primary treatment modality	
Surgery	233 (89.3)
Lobectomy	176 (75.5)
Segmentectomy	13 (5.6)
Wedge resection	42 (18)
Pneumonectomy	2 (0.9)
External Beam Radiation	8 (3.0)
SBRT	16 (6.1)
Radiofrequency Ablation	3 (1.1)
Cryoablation	1 (0.4)
Adjuvant chemo	
Yes	6 (2.3)
No	255 (97.7)
Pathologic diagnosis	
Adenocarcinoma	173 (66.3)
Squamous	67 (25.6)
Adenosquamous	4 (1.5)
Pleomorphic carcinoma	3 (1.1)
Large Cell Neuroendocrine	1 (0.4)
Carcinoma, NOS	2 (0.8)
Sarcomatoid	1 (0.4)
Unknown	10 (3.4)
Grade	
Well differentiated	44 (16.9)
Moderately Differentiated	99 (38.0)
Poorly differentiated	70 (26.8)
Undifferentiated	6 (2.3)
Unknown	42 (16.1)

### Clinical features of recurrence and SPLC

With a median follow-up of 76 months, there were 65 recurrences (24.9%) and 31 SPLC (11.9%) (**Table 2**). Sixty (89.2%) patients had recurrence of disease within 5 years, and 7 (10.8%) patients had recurrence of disease between 5 to 10 years. There were no recurrences beyond 10 years. For SPLC, 16 (51.6%) occurred within 5 years, 11 (35.5%) occurred between 5-10 years, and 4 (12.9%) occurred beyond 10 years. Locoregional recurrence occurred in 30 patients (46.1%), and distant recurrence occurred in 25 patients (53.8%). For SPLC, adenocarcinoma was the most common pathologic diagnosis (n=19; 61.3%). Of the 26 patients who were never-smokers and diagnosed with stage 1A disease, only 1 (3.8%) had SPLC and 1 had recurrence (3.8%). Of the 45 patients with smoking history and stage 1B disease, 7 (15.5%) developed a SPLC and 18 had a recurrence (40%).

**Table 2:** Clinical features of 65 recurrences and 31 SPLC.

Characteristic	Recurrence (n=65)	SPLC (n=31)
	No (%)	No (%)
Time to Occurrence		
Within 5 years	60 (89.2)	16 (51.6)
5-10 years	7 (10.8)	11 (35.5)
Beyond 10 years	–	4 (12.9)
Location		
Locoregional	30 (46.1)	31 (100)
Distant	35 (53.8)	–
**Treatment**		1 (3.5)
Chemo	6 (9.2)	6 (22.6)
RT	16 (24.6)	11 (35.5)
Surgery	3 (4.6)	4 (12.9)
Chemo +RT	23 (35.4)	–
Chemo + Surgery	1 (1.5)	–
Chemo + RT + Surgery	2 (3.1)	1 (3.2)
Surgery + RT	2 (3.1)	1 (3.2)
Ablation	1 (1.5)	3 (9.8)
Unknown	6 (9.2)	3 (9.8)
None	5 (7.7)	
**Pathologic diagnosis**		19 (61.3)
Adeno	40 (61.5)	5 (16.1)
Squamous	16 (24.6)	1 (3.2)
Adenosquamous	1 (1.5)	3 (9.7)
Small Cell	–	–
Pleomorphic carcinoma	1 (1.5)	–
Carcinoma, NOS	1 (1.5)	–
Sarcomatoid	1 (1.5)	3 (9.7)
Unknown	5 (7.7)	

### Clinical factors association with DFS and recurrence

**Figure 1:**
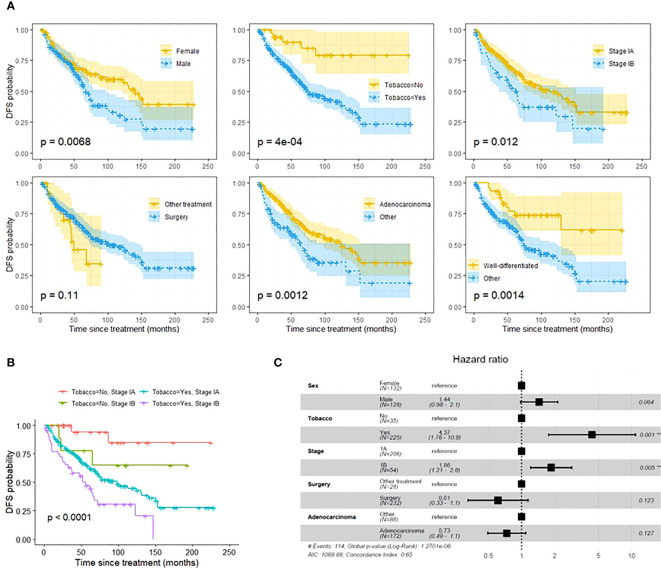
**(A)**: Univariate DFS analysis using Kaplein-Meir method. **(B)**: DFS analysis using Kaplan-Meier method demonstrating that a combination of both stage IB tumors and smoking history are associated with worse DFS than one risk factor or no risk factors. Pathologic stage and smoking status were selected based on multivariate DFS composite risk analysis (see **Figure 2**). **(C)**: Multivariate DFS composite risk analysis demonstrating tobacco use and stage IB tumors are associated with significantly decreased DFS. Tumor grade was excluded from analysis due to 42 missing values. **p<0.05, #, Number.

**Figure 2:**
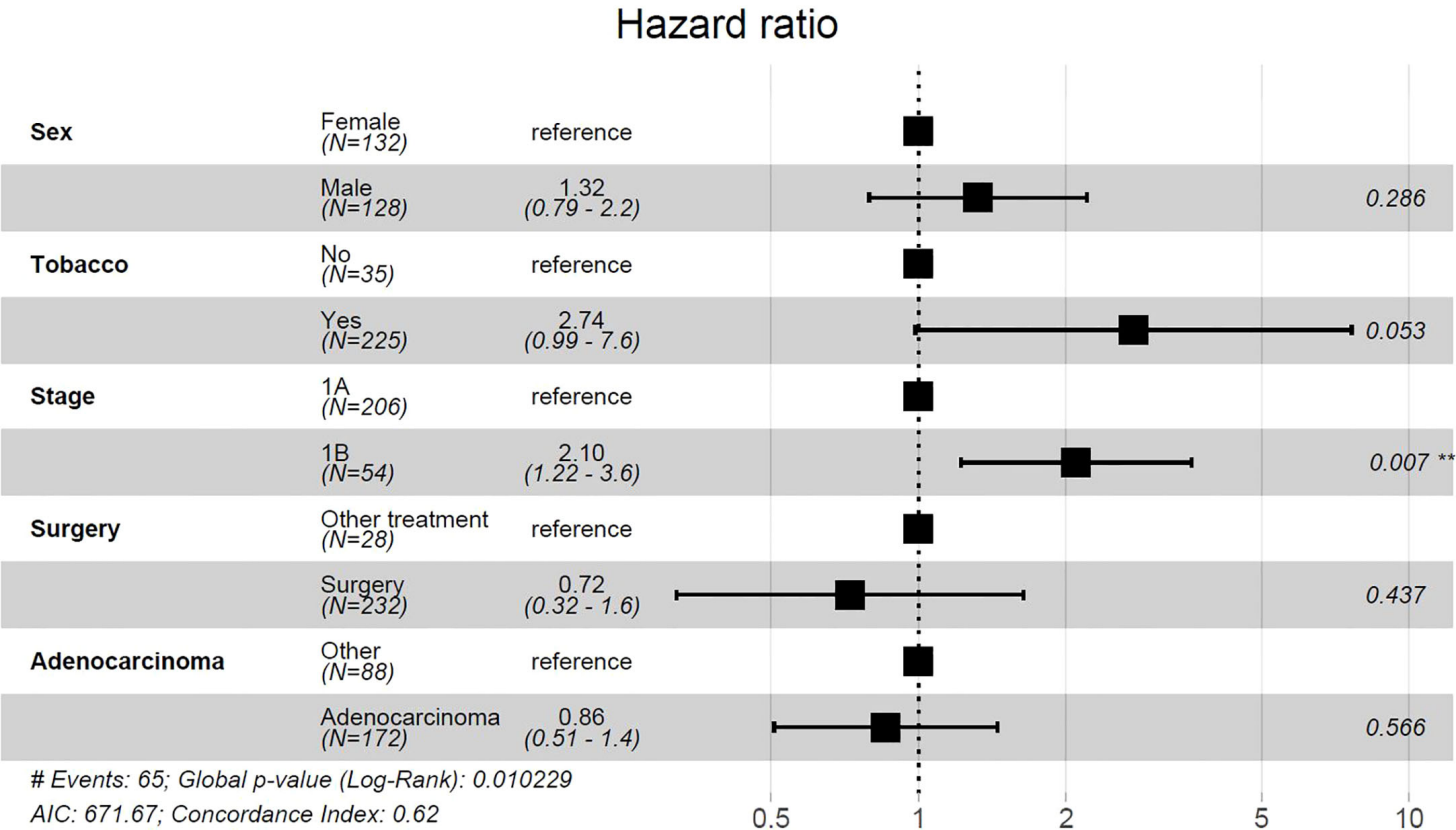
Multivariate cox proportional-hazards model for risk of NSCLC recurrence. Tumor grade was excluded from analysis due to 42 missing values. **p<0.05, #, Number.

On univariate analysis, female gender, smoking history, stage IB tumors, non-adenocarcinoma histology, and non-well differentiated histology were associated with a significantly decreased DFS (**Figure 1A**). Treatment modality was not associated with DFS difference. After adjusting for covariates, multivariate analysis showed that tobacco use and stage IB tumors were associated with significantly decreased DFS (p=.001 and p=.005) (**Figure 1C**). Patients with both smoking history and stage IB had further decreased DFS compared to patients with only one risk factor or no risk factor (p<0.0001) (**Figure 1B**). Among 26 patients who were non-smokers and diagnosed with stage 1A tumor, only 1 patient (3.8%), developed a recurrence within 5 years. Univariate cox proportional-hazards model identified tobacco use and stage 1B tumors as factors that were associated with a significantly increased risk of recurrence (**Supplemental Table 1**). After adjusting for covariates, multivariate cox proportional-hazards model showed stage IB associated with significantly increased risk of NSCLC recurrence (**Figure 2**). Stage IB tumors had a hazard ratio (HR) of 2.1 (CI 1.22-3.6; p=0.007).

## Discussion

Cancer recurrence following curative surgery for stage I NSCLC has been reported, ranging from 14-36% (7, 9). However, the optimal duration for surveillance CT scans remains unknown. Although the low dose CT scan is considered relatively safe, the cumulative dose of radiation exposure over a long period of time still carry risks of secondary malignancy (10). Several previously reported incidences of developing recurrence is around 20% of patients (5). In our study, 25% of patients developed a recurrence, slightly higher than previously reported data. Consistent with the previous studies, we demonstrated that most recurrences are within the first 5 years (5). Our study identified smoking and stage IB disease as factors associated with decreased DFS, and stage IB tumors were also associated with overall risk of recurrence after controlling for gender, smoking status, histologic subtype, and treatment modality. Chen et al’s study similarly demonstrated increased risk for local regional recurrence with advanced pathological stage (6). However, different from our study, Chen’s study identified tumor grade/differentiation as the only significant risk factor for recurrence in multiple logistic regression analysis. Tumor grade was not reported in 42 of our patients, thus potentially limiting our analysis. Our study showed that patients who are never-smokers and diagnosed with stage IA lung cancer may not benefit from surveillance CT scans after 5 years, indicating a personalized surveillance approach should be considered. Due to limitations of population size and lack of racial diversity, studies with larger patient numbers and racial diversity are warranted to draw more robust conclusions.

Another consideration is the risk of subsequent development of SPLC. The incidence of SPLC has been estimated at approximately 1 to 2% per year, with the cumulative risk increasing over time (11, 12). In a retrospective review of patients in the Surveillance, Epidemiology, and End Results (SEER) database, the median time interval of SPLC diagnosis was between 59 and 62 months (12). Our study showed that 16 patients developed a SPLC within 5 years, and 15 patients developed a SPLC following 5 years. In the lower risk population of patients with stage IA disease and non-smokers, only 1 patient (3.8%) developed a SPLC. A more extended follow up period may be warranted for monitoring of SPLC, especially in those higher risk patients.

## Conclusion

CT imaging for surveillance beyond 5 years may not be necessary in low risk patients. Our study found that in patients who were non-smokers and diagnosed with stage IA disease, there was a relatively low risk of recurrence or development of SPLC after five years. An individualized approach, which takes into account risk factors of smoking history and pathologic stage of IB, may be useful in determining whether to continue annual CT surveillance indefinitely following curative therapy for stage I NSCLC.

## Data availability statement

The original contributions presented in the study are included in the article/**Supplementary Material**. Further inquiries can be directed to the corresponding author.

## Ethics statement

The studies involving humans were approved by Mayo Clinic Institutional Review Board. The studies were conducted in accordance with the local legislation and institutional requirements. Written informed consent for participation was not required from the participants or the participants’ legal guardians/next of kin. No consent is required for this type of retrospective chart review study.

## Author contributions

EB: Investigation, Writing-Original Draft, Visualization, Project administration. DG-B: Investigation, Writing- Review and Editing. TP: Investigation, Writing- Review and Editing. MM: Formal analysis, Writing-Review and Editing. FH: Methodology, Formal analysis, Writing-Review and Editing, Visualization. RC: Formal analysis, Writing-Review and Editing. YZ: Investigation, Writing- Review and Editing. RM: Investigation, Writing-Review & Editing. YL: Conceptualization, Methodology, Investigation, Writing-Review and Editing, Supervision. All authors contributed to the article and approved the submitted version.
