# Supplementary material for: Brief report: risk stratification following curative therapy for stage I NSCLC
Source: Front Oncol. 2023 Aug 14;13:1250315. doi: 10.3389/fonc.2023.1250315 (PMC10461311; doi:10.3389/fonc.2023.1250315)
Supplement: Supplementary file 1 [file Table_1.docx]

|  | | Unadjusted HR | P-value |
| --- | --- | --- | --- |
| Gender | Female | Reference | -- |
|  | Male | 1.5021 | 0.106 |
| Tobacco* | No | Reference | -- |
|  | Yes | 2.7878 | 0.0471 |
| Stage | IA | Reference | -- |
|  | IB | 1.9922 | 0.00938 |
| Treatment | Others | Reference | -- |
|  | Surgery | 0.7746 | 0.525 |
| Histologic subtype | Adenocarcinoma | Reference | -- |
|  | Others | 1.5233 | 0.0994 |
| Grade** | Well differentiated | Reference | -- |
|  | Others | 1.9139 | 0.09 |
| *1 observation deleted due to missing data | | | |
| **42 observations deleted due to missing data | | | |

Supplemental Table 1: Univariate Cox proportional-hazards model for risk of NSCLC recurrence
